# A Case in Surgery

**Published:** 1888-08

**Authors:** R. H. Taylor

**Affiliations:** Griffin, Ga.


### A CASE IN SURGERY.*

BY R. H. TAYLOR, M. D., GRIFFIN, GA.

In writing this report my chief object is to call attention to the fact that we cannot be too careful as physicians in making our diagnoses, even in those cases of seeming slight consequence.

It is too much a habit, I fear, with a goodly number of the profession to take too many things for granted, and, I may say, accept the diagnosis given by the patient himself without sufficient inquiry and investigation for ourselves.

I cannot better illustrate my meaning than by giving a brief report of a case that came under my observation since the last meeting of our Association:

F. M. S., white man, aged about 33 years; occupation, farmer, and very muscular. Family history good, and no evidence or history of any specific disease. About eight or ten years ago he made complaint to his family physician that he was suffering

*Read by title—Thirty-Ninth Annual Meeting of the Medical Association of Georgia.

with "piles." Upon this statement, without any investigation of his own, he, the family physician, proceeded to fix up a "pille ointment," and thus the case was treated for several months without the doctor's making any examination whatever of his patient's anus or rectum. Of course the case continued to grow worse and he began to tell his neighbors how much he had suffered with piles and Doctor ——— could do nothing for him. Now this kind of intelligence seems to have a peculiar way of its own of disseminating itself, and it was not long before he was flooded with pamphlets and advertising sheets, in big letters, "Sure Cure for Piles," "No Cure, No Pay," "Consultation Free," and all that sort of thing.

As a matter of fact, however, he took in the bait. He had suffered so much and his physician had done him no good, and then "the man talks so fair there can be no harm in trying." But to cut the matter short, he went and was fleeced by the mountebank, and was told that his piles were cured; but, alas, his relief was destined to be short-lived, and his great doctor gone with his money, and no trace left as to his whereabouts. Thus the case came into my hands.

When I first saw him and made an examination, I found two or three fissures, perfectly typical, but very deep and long, with the most tightly contracted sphincter muscle I ever saw. The size of the muscle was unusually large and at once attracted my attention. On attempting to introduce my finger into the rectum, I met with such a vigorous spasm of this muscle that I at once ceased all further effort in that direction.

There was a small protuberance on the verge of the anus that felt hard as cartilage. Just on the inside of this teat-like projection I found the opening of a small sinus, which the probe proved to be running back, away from the rectum, in the direction of the tip of the coccyx, to the extent of probably three-quarters of an inch, and ended in a small pouch or abscess sack.

Pressure on this sack caused a drop or two of pus to start from the opening of the sinus in the verge of the anus.

I gave him ether and introduced both thumbs into the rectum, and as gently as possible, for it took all my strength, so powerful was the sphincter, completely stretched the muscle until the inside of my thumbs rested against the tuber ischii. I then opened up the sinus to the bottom and scraped out all soft granulations and the entire abscess sack. After paralyzing the sphincter muscle I examined the inside of the rectum and found the mucous membrane perfectly smooth and healthy. I could find no evidence of the fact that he had ever had hemorrhoids, although his "pile doctor" had assured him that he had removed all his "pile tumors."

Now, had his family physician examined his anus and rectum half as carefully as he would have done his lungs ; had he complained to him of symptoms pointing to disease of the respiratory organs, he would have seen at once his error, and would either have operated himself and cured his patient, or sent him to some intelligent surgeon who would have done so. Now, may we not ask ourselves if we are not largely responsible for the existence of many quacks who travel all over the country and humbug the people after this fashion?

A little care in making a clear diagnosis in this case would have saved both time, money and suffering to the patient and reputation to the physician. Therefore we cannot urge too strongly thorough investigation and careful examination of every case, however trivial it may at first appear.

I cannot close this report without calling attention to an accident that befell me in the operation just reported.

When my patient was coming from under the influence of the ether, the retching and straining caused a prolapsus of the rectum, the replacing of which gave my patient considerable pain. Had I taken the precaution to place a compress over the anus, and held it firmly in place with a T bandage immediately after the operation, I would have saved my patient considerable pain and myself the self-reproach of not being more careful.

The compress and the T bandage were placed on as soon as the prolapsus was reduced, and worn with great comfort for four or five days. I found that this simple measure relieved all that uneasiness and sense of weight or bearing down in the rectum that I have found so constant after this operation, in many cases requiring considerable quantities of morphine to control.

Pursuing the same line of thought, we might go still farther and make a more extended application of the principles involved in the treatment of the foregoing case.

In view of the importance of the treatment of old abscesses with fistulous openings, and, in fact, old sinuses of every description, and in any part of the body they may be located, I may venture to trespass a little more on your time.

In the treatment of this class of cases the powerful influence of modern antiseptic surgery is well illustrated. To those who have not seen for themselves the results of thorough antisepsis and scrupulous cleanliness in these operations, it wlll scarcely seem credible to tell them that the deepest of these sinuses may be laid freely open to the *very bottom* all fungus granulations, and every part of abscess sacks thoroughly scraped out with a sharp spoon, the wound filled with antiseptic gauze and forced to heal from the bottom by granulation, and that, too, without any considerable pain or inflammation following, and scarcely a drop of pus be discharged during the entire process; but such is even the case.

To obtain such results we must be scrupulously clean and careful, and every instrument, no matter how perfect it may appear, must be treated just as if we saw whole hosts of bacteria and myriads of micrococci clinging to every particle of available space on its shining surface. All sponges, surgeon's and assistant's hands, and in fact everything that comes in contact with the wound, must be treated in the same way, never forgetting to make a free use of soap and water before using any antiseptic solution.

One point I would especially impress, and that is, *partial* incisions and the injections of iodine, carbolic acid, etc., is but a waste of time.

Never stop short of a free opening to the *very bottom*. I might write on, page after page, detailing the minutiæ of antisepsis; but with the abundance of literature on this subject, it is not necessary.

Suffice it to say that the best results from the use of antiseptic fluids can only be expected when preceded by thorough cleansing with soap and water.

